# Yeasts in different types of cheese

**DOI:** 10.3934/microbiol.2021027

**Published:** 2021-11-08

**Authors:** Thomas Bintsis

**Affiliations:** Collaborating Teaching Staff at Hellenic Open University, Greece

**Keywords:** cheese yeasts, hard, semi-hard, soft, white brined, mould surface ripened, bacterial surface ripened, blue cheese

## Abstract

Yeasts constitute an important part of cheeses, and especially the artisanal ones. The current study reviews the occurrence of yeasts in different cheese varieties and the role of yeasts in cheesemaking process. The use of molecular methods for identification and strain typing has extended the knowledge for yeast diversity in cheeses. For the study of the occurrence of yeasts in different cheese types, seven categories are used, that is: 1) hard, 2) semi-hard, 3) soft, which includes soft pasta-filata and whey cheeses, 4) white brined cheeses, 5) mould surface ripened, 6) bacterial surface ripened cheeses, and 7) blue cheeses. For some cheese types, yeasts are the main microbial group, at least for some part of their ripening process, while for some other types, yeasts are absent. Differences between industrially manufactured cheeses and artisanal cheeses have specified. Artisanal cheeses possess a diverse assortment of yeast species, mainly belonging to the genera *Candida*, *Clavisporalus*, *Cryptococcus*, *Debaryomyces*, *Geotrichum*, *Issatchenkia*, *Kazachstania*, *Kluyveromyces*, *Kodemaea*, *Pichia*, *Rhodotorula*, *Saccharomyces*, *Saturnispora*, *Torulaspora*, *Trichosporon*, *Yarrowia* and *ZygoSaccharomyces*. The role of the yeasts for selected cheeses from the seven cheese categories is discussed.

## 1. Introduction

Cheese has been produced and consumed for thousands of years. There are as many as 1500 cheese varieties identified around the world; every variety displays specific sensory characteristic and thus displaying a diversity of cheeses with different quality characteristics, such as appearance, flavor, aroma, and texture [1]. These cheeses are well-adapted to the local conditions of the environment as well as the cheesemakers' knowledge and social position. Either with the aid of acid, or keeping the milk into the stomachs of slaughtered young animals, cheesemaking was first based on the microflora of raw milk and the “inoculation” of the milk with a sample of a previous day product, i.e. back-slopping.

Up to the 20th century, cheesemaking remained an unregulated process. The introduction of pasteurization and the discovery and characterization of lactic acid bacteria have changed the views on the way cheese is manufactured [2]. In the early 1960s, commercial starter culture were developed for direct vat inoculation. Nowadays, cheesemaking has progressed to be a fully automated process and requires large quantities of milk, and total control of the process, the use of pasteurized milk and commercial starter cultures for a standardized and successful production of any cheese variety. It should be noted that both traditional or artisanal and industrial cheeses are manufactured following the same basic steps for cheese-making, depending on the cheese type. The industrial ones are standardized, with consistent quality, deliver all the cheese's nutrients nutritious and offer convenience at an economical price to the main group of consumers [3]. Instead, the traditional or artisanal cheeses are locally produced, usually craft-made and using the milk of one, or limited number of farms; these cheeses have a strong linkage to the territory of origin (i.e., climate, landscape, rural development and human knowledge and resources) and therefore are testimonial of the history, the culture and the agricultural life of the local cheesemaker's communities [3]. Organoleptic differences between cheeses are obvious from a great part of consumers, with the industrially manufactured cheeses have recognized by part of the consumers, being bland and uninspiring, and artisanal cheeses have gained a great proportion of sales [4]. Thus, a new group of artisanal cheeses have been developed; these are manufactured following the principles of traditional way, on small scales, but which often employ advanced practices and techniques that satisfy the updated international public health regulations; at the same time preserving the traditional cheesemaking process [4]. Traditional cheeses collectively offer a rich diversity of intrinsic physicochemical and organoleptical characteristics [3]. Many of the special characteristic are partly attributable to the enriched and diverse microfloras of many traditional cheeses [5].

A variety of artisanal cheeses are manufactured from raw milk, and raw milk's microbiota is an important part final cheeses' microbiota [5]. It is generally accepted that cheese made from raw milk matures in a different way and develops a more intense flavour than that made from pasteurized milk. The main characteristics of raw milk cheeses is that the manufacturing and the maturation is driven by a complex microbial community. Thus, microbial communities appear to be a key player in the development of cheese quality properties. In addition, in terms of safety, raw milk microbial communities may act as a bio-preservative shield against microbial pathogenic and spoilage populations [6]. Recently, a number of microbial cheese diversity studies, that combine both phenotypic and genotypic approaches have published, revealing the complexity of such communities [7],[8]. The distinct microbial consortia from the processing environment have developed an “in house” microflora, which is identical to the specific dairy facility, and this is, possibly, explaining the added diversity of cheese characteristics expressed by each cheese variety [9].

Yeast are eukaryotes, that is, they contain an identifiable nucleus and most of yeasts contain chitin, which is responsible for their rigid structure [10]. Yeasts are not nutritionally demanding microorganisms and, comparing with bacteria, are larger and grow more slowly; thus, yeasts do not compete with bacteria. However, yeasts grow well at acidified environments, where bacteria either do not grow or grow only very poorly. The low pH of freshly made cheese is therefore partially selective for their growth, against bacteria.

Sexual reproduction of yeasts is named ‘teleomorph’, and is considered as the perfect form, while asexual reproduction, that is ‘anamorph’, is considered as the imperfect form. Taxonomically, the teleomorphic name is used, however, in one case, the anamorphic name *Geotrichum candidum* is used, and this will be used in the present paper, rather than *Galactomyces candidum*, which is the teleomorphic [10].

Most of the identified microorganisms present in raw milk are lactic acid bacteria and the importance of these microbes in cheese ripening is well recognized [11]. However, the yeast population is also important [12]–[15] and is associated with the secondary microbiota of a number of cheeses, mostly the artisanal ones, where they have an impact on the maturation process.

The total raw milk yeast counts are generally in the range of 10–10^3^ cfu/mL [16], and yeast genera commonly identified in raw milk include *Candida*, *Cryptococcus*, *Debaryomyces*, *Geotrichum*, *Kluyveromyces*, *Trichosporon*, *Pichia* and *Rhodotorula* spp. [12]–[15]. *Candida rugosa*, *G. candidum, Torulaspora delbrueckii* and *Yarrowia lipolytica* were common yeast species found in raw milk [17]–[20]. Büchl and Seiler in their excellent review on yeasts in milk and dairy products reported 21 yeast species in milk, with *G. candidum*, *Issatchenkia occidentalis*, *Issatchenkia orientalis*, *Kluyveromyces marxianus*, *Pichia anomala*, *Pichia fermentans* and *Trichosporon beigelii* being the most frequently found [20].

Commercial ripening yeast cultures have been developed for special cheese types [21]; these include the selected strains of *G. candidum* in the production of mould surface ripened cheeses [22]; the lactose-fermenting species *K. marxianus*, *Kluyveromyces lactis*, *Saccharomyces cerevisiae* and *Debaryomyces hansenii* in mould surface ripened and blue cheeses [7],[16],[23].

The aim of the current study is to review the occurrence of yeasts in different cheese varieties and thus study the role of yeasts in cheesemaking process. For this reason, the great variety of cheese types is categorized into seven categories, that is: 1) hard (moisture content less than 43%), 2) semi-hard (moisture content of 44–55%), 3) soft (moisture content more than 56%), which includes soft pasta-filata and whey cheeses, 4) white brined cheeses, 5) mould surface ripened, 6) bacterial surface ripened cheeses, and 7) blue cheeses. For certain cheese types that excluded from the categorization, yeasts have not reported to be an important part of their microflora.

## 2. Methods for identification and typing

Yeast identification in milk and milk products was traditionally carried out using the characteristics of the colonies, microscopy, and phenotypical characteristics, such as growth requirements, and assimilation and/or fermentation of certain carbohydrates and nitrogen compounds [24],[25]. However, these methods are laborious, complex and may give confusing results [26]. Throughout the last 20 years, yeast identification is based on sequencing of the D1/D2 region of the 26S rRNA gene and the internal transcribed spacer (ITS) domains (ITS1 and ITS2) divided by the conserved 5.8S rRNA gene [26]–[30].

Denaturing High-Performance Liquid Chromatography (DHPLC) has been applied for the identification of yeasts in bacterial surface ripened cheeses [31] and for the assessment of microbial diversity in natural whey cultures used for the manufacture of an Italian pasta-filata cheese [32]. Recently, advanced methods such as Matrix Assisted Laser Desorption Ionization-Time of Flight Mass Spectrometry (MALDI-TOF MS) and Fourier Transform Infrared Spectroscopy (FTIR) have applied for dairy yeast identification purposes [24],[33]. MALDI-TOF MS generates protein-based spectral profiles, that is fingerprints acquired by desorption of specific peptide/protein biomarkers released from the cell surface by acidic treatment, while FTIR is based on the detection of functional biochemical groups directly from intact cells, producing metabolic spectral fingerprints unique for yeast species [24],[34]. Quigley et al. reviewed the application of molecular methods such as DHPLC, Temporal Temperature Gradient Gel Electrophoresis (TTGE) and Single Stranded Confirmation Polymorphisms (SSCP) for the identification of microbes, including yeasts, in dairy products from raw milk [35]. The SSCP method has been used for the evaluation of yeast diversity in Salers cheese, a raw milk cheese stored in a wooden container [36].

Strain typing is essential to trace the yeasts in the dairy environment in order to study its microbial ecology. Yeast genotyping and cluster analysis of the DNA fingerprints are often introduced prior DNA sequencing [24]. Pulsed-Field Gel Electrophoresis (PFGE) is commonly used to evaluate intraspecies diversity of chromosome arrangements or chromosome-length polymorphism [37] and have frequently applied for *D. hansenii* and *K. marxianus* strains from cheeses [38]–[40]. In addition, Randomly Amplified Polymorphic DNA (RAPD), employing a single primer M13 for random amplification of complementary genome sequences, was used for yeast classification in cheese [19] and for typing of *D. hansenii* strains [41]. Multilocus Sequence Typing is a popular yeast typing method and has been applied to the typing of *K. marxianus* and *D. hansenii* isolated from different types of traditional French cheeses such as Camembert, Chevrotin des Aravis, Saint-Nectaire and from Spanish Roncal cheese [42]. The same method has recently applied for the typing of *G. candidum* isolated from starter cultures and cheeses [43].

Since the culture-dependent methodologies require isolation on selective media, a more comprehensive overview on both viable and dead microorganisms can be obtained by culture-independent techniques and advanced techniques such as pyrosequencing or Illumina sequencing have been used [44]–[46].

The molecular approaches based on the use of metagenomics combined with high-throughput sequencing are currently used in order to profile dominant and subdominant microbial populations on a large scale [41]. Wolfe et al. [48] and Quigley et al. [49] used such approaches to reveal the cheese rind microbiota of certain artisanal cheeses and concluded on the correlation between cheese characteristics with the composition of cheese microbial community at different stages [49].

## 3. Occurrence of yeasts in cheeses

### 3.1. Hard cheeses

Hard cheeses are cheeses with moisture content less than 40% and, usually, a long maturation process (up to 30 months); they are characterized by a granular texture and a strong flavor. Natural whey cultures, thermophilic or mesophilic starters are used for the acidification, and the coagulum is cooked at high temperatures [50]. The most important yeasts isolated from hard cheeses are shown in Table 1.

Fleet and Mian [51] reported that Australian Cheddar cheeses contained yeast counts of 10^4^–10^6^ cfu/g. Similarly, Welthagen and Vijoen [52] reported that the yeasts counts were varied from 10^2^ to >10^7^ cfu/g in South African Cheddar; additionally, 88% of the cheeses had 10^5^ cfu/g, a level deemed necessary to influence flavour development. During ripening, the density increased from 10^2^ to 10^3^ cfu/g over the first 30 days of ripening, later increased to 10^6^ cfu/g, and then, towards the end of maturation decreased again [52].

**Table 1.** Yeast species isolated from hard cheeses

Yeasts species	Cheese	Reference
*Candida catenulate*	Canestrato Pugiese	[60]
*Candida etchellsii*	Cotija	[58]
*Candida glaebosa*	Pecorino di Farindola	[53]
*Candida lambica*	Fiore Sardo	[19]
*Candida parapsilosis*	Pecorino di Farindola	[53]
*Candida zeylanoides*	Fiore Sardo, Pecorino di Farindola	[19],[53]
*Debaryomyces hansenii*	Fiore Sardo, Pecorino Romano, Serro Minas	[19],[55],[59]
*Geotrichum candidum*	Fiore Sardo	[19]
*Kluyveromyces lactis*	Fiore Sardo, Pecorino Crotonese	[19],[54]
*Kluyveromyces marxianus*	Pecorino di Farindola, Pecorino Romano, Serro Minas	[53],[55],[59]
*Kodamaea ohmeri*	Serro Minas	[59]
*Pichia kudriavsevii*	Cotija, Pecorino di Farindola	[53],[58]
*Rhodotorula* spp.	Pecorino Romano	[55]
*Saccharomyces cerevisiae*	Pecorino Romano	[55]
*Trichosporon cutaneum*	Canestrato Pugiese	[60]
*Yarrowia lipolytica*	Canestrato Pugiese, Pecorino Crotonese	[54],[60]

Pecorino di Farindolais an Italian hard raw milk cheese locally made by farmers, and Tofalo et al. [53] evaluated its yeast consortia during cheesemaking and maturation. Yeast counts ranged from 10^5^ cfu/g at sale to 10^4^ cfu/g for the matured cheese. Similar values have been reported for Pecorino Crotonese [48]. Using molecular identification by a combination of PCR-RFLP of the 5.8S ITS rRNA region and sequencing of the D1/D2 domain of the 26S rRNA gene, *K. marxianus* was the predominant species. Yeast species such as *Pichia kudriavzevii*, *Candida parapsilosis*, *Candida glaebosa* and *Candida zeylanoides* were present only during the early weeks of ripening. *D. hansenii*, *K. marxianus*, *Saccharomyces cerevisiae* and species of *Rhodotorula*, dominated Pecorino Romano, as well as Pecorino di Filiano [55]–[57].

The autochthonous microbiota of Cotija, a traditional extra-hard Mexican raw milk cheese were studied during 90 days of maturation under traditional and controlled conditions [58]. Using molecular assessments by PCR-DGGE 26S and 16S rRNA encoding regions, a complex microbial profile was found, and *Candida etchellsii, Pichia kudriavsevii* and *Moniliella suaveolens* were found in the cheese matrix [58].

The yeast populations in Serro Minas cheese, a traditional and popular cheese produced from raw milk in Brazil, were studied over the course of 60 days of maturation [59]. Enzymatic activity exhibited by these yeast isolates was also studied. A total of 19 yeast species were identified, and the predominant yeasts included *D. hansenii*, *Kodamaea ohmeri* and *K. marxianus*. *D. hansenii* showed low lipolytic and high proteolytic activity. *K. marxianus* demonstrated lipolytic and β-galactosidase activity and *K. ohmeri* displayed low lipolytic and β-galactosidase activity.

Corbo et al. [60] investigated the yeasts from typical Apulian cheeses from Italy, aiming to further select appropriate starter cultures for cheese production. The most prevalent isolates from Canestrato Pugiese belonged to the species *Trichosporon cutaneum*, *Candida catenulate* and *Yarrowia lipolytica* [60].

The presence and the role of yeast microbiota was investigated in artisanal Fiore Sardo cheese throughout the maturation and strains belonging to the prevalent species *D. hansenii*, *K. lactis*, *G. candidum*, *Candida zeylanoides* and *Candida lambica* were selected for technological and genotypic characterization [19]. They reported that *D. hansenii* strains fermented glucose and assimilated lactate, while some strains showed the ability to assimilate citrate. As far as the emzymic activities, only a few *D. hansenii* strains showed proteolytic and lipolytic activity. *K. lactis* was able to both assimilate and ferment lactose, to assimilate lactate but not citrate and to show proteolytic but not lipolytic activity. *G. candidum* assimilated lactate and some strains showed proteolytic and lipolytic activity. *C. zeylanoides* assimilated lactate and citrate and showed lipolytic activity and *C. lambica* fermented glucose and assimilated lactate.

Interestingly, selected yeasts such as *G. Geotrichum*, *Pichia jadinii, Y. lipolytica* and *D. hansenii* were studied for lactic acid utilization, lipolysis, proteolysis and flavour development in foil ripened Raclette cheeses [61]. Throughout the maturation, the lactic acid content was increased, probably as a result of increased lactic acid bacteria, and yeast metabolites may have a positive contribution. Yeasts showed either esterase or lipase activity. Moreover, yeasts revealed peptidase activity, and an increase in small peptides and free amino acids was observed. *Y. lipolytica* was capable of improving the overall sensory characteristics of cheese, but *G. Geotrichum*, *Pichia jadinii* and *D. hansenii* had a neagative effect on the organoleptic properties of the final cheese.

### 3.2. Semi-hard

The category of semi-hard cheeses is a heterogeneous cheese category. Semi-hard cheeses have a moisture of 44–55%, and there are semi-hard cheeses which could belong to more than one category, e.g., Gouda cheese, which belong to Dutch-type cheeses. Some of them may be consumed as semi-hard, or at a longer maturation period, as hard cheese [50]. The most important yeasts isolated from semi-hard cheeses are shown in Table 2.

The yeast microflora was studied for an artisanal semi-hard cheese made from raw ovine milk manufactured in South Portugal by Pereira-Dias [62]. A total of 344 yeasts strains were isolated from the curd and cheese body during the 60 days maturation. Esterase activity was common to most of the isolates, while proteolysis was observed in only a few of them. *D. hansenii* and *Candida intermedia* were the most frequent species and these two species increased to 86% at the end of the maturation. Padilla et al. [41] used molecular methods for the identification of yeast from four ovine and caprine raw milk semi-hard cheeses, produced in a small dairy sited within the borders of the Natural Park Sierra de Espadán, Castellón, Spain. Yeast counts of ovine milk cheeses, started at 10^4^ and 10^5^ cfu/g and increased to 10^7^ cfu/g at the third week of the maturation, while for caprine milk cheeses, the yeast counts started at 10^4^ and 10^5^ cfu/g reaching 10^8^ cfu/g, at the end of maturation. *D. hansenii* and *K. lactis* isolates were found to be the most abundant yeast species, and other yeast species were isolated in minor numbers. In all cheeses, yeast diversity decreased along the cheese maturation. The yeast isolates were also studied for several technological features and most yeast isolates showed proteolytic activity.

Canastra cheese is a semi-hard cheese which is produced in seven municipalities in the state of Minas Gerais in Brazil. This cheese is produced from bovine raw milk inoculated with the commercial rennet and pingo, which is a type of natural starter obtained from the cheese whey from the previous day [33]. Thirty nine isolates capable of fermenting lactose in a synthetic medium were identified by MALDI-TOF as *K. lactis*, *T. delbrueckii* and *C. intermedia*. Borelli et al. [63] reported *Kodomaeda ohmeri*, *D. hansenii*, *T. delbrueckii* and *K. lactis* as the most frequent yeasts in Canastra cheese. *K. lactis* is frequently isolated from dairy products such as cheeses, which might be due to its capacity of fermenting lactose.

**Table 2.** Yeast species isolated from semi-hard cheeses

Yeasts species	Cheese	Reference
*Candida intermedia*	Semi-hard ovine cheese, Canastra	[33],[62]
*Candida parapsilosis*	Semi-hard ovine and caprine cheese	[41]
*Candida sake*	Fontina	[67]
*Candida zeylanoides*	Semi-hard ovine cheese	[62]
*Clavispora lusitaniae*	Tomme d' Orchies	[65]
*Debaryomyces hansenii*	Semi-hard bovine, ovine and caprine milk cheeses, Canastra, Fontina, Tomme d' Orchies	,[41],[62],[65],[66],[67][69]
*Kazachstania unispora*	Semi-hard bovine and caprine cheese	[41]
*Kluyveromyces lactis*	Semi-hard bovine, ovine and caprine cheese, Canastra, Tomme d' Orchies	[33],[41],[65],[66],[69]
*Kluyveromyces marxianus*	Tomme d' Orchies	[65]
*Kodomaea ohmeri*	Canastra	[69]
*Pichia fermentans*	Semi-hard bovine cheese	[66]
*Pichia guilliermondii*	Semi-hard bovine cheese	[66]
*Saccharomyces cerevisiae*	Semi-hard bovine cheese	[66]
*Saturnispora mendoncae*	Tomme d' Orchies	[65]
*Torulaspora delbrueckii*	Canastra	[33],[69]
*Yarrowia lipolytica*	Semi-hard bovine, ovine and caprine cheese, Tomme d' Orchies	[41],[65],[66]

Sepra cheese is a Portugese artisanal cheese produced within the Alentejo province from raw sheep's milk, using rennet produced from the dried flowers of *Cynara cardunculus* L. and without the addition of a starter culture [64]. *Debaryomyces* spp. and *Kluyveromyces* spp. were the predominant genera. In addition, although in one sample, *Kluyveromyces* spp. was the dominant yeast in spring cheese, while in winter cheese its abundance was lower than *Candida* spp. Gonçalves Dos Santos et al. [64] demonstrated that the yeast community of Serpa cheese is composed of a wide diversity of species and they play an important role in the development of the sensory characteristics of the final cheese. Ceugniez et al. [65] studied the yeast diversity in Tomme d' Orchies, a French artisanal cheese, from the North of the country. A great diversity in yeast microflora and species were identified as *D. hansenii*, *K. lactis*, *K. marxianus* and *Y. lipolytica*; infrequent species were identified as *Clavispora lusitaniae* and *Saturnispora mendoncae*.

Atanassova et al. [66] identified by both genotyping and sequencing methods: *Y. lipolytica*, *K. lactis*, *D. hansenii*, *Pichia guilliermondii*, *P. fermentans* and *S. cerevisiae* from short ripened starter-free raw bovine milk cheese, made in Galicia, in Spain. *Y. lipolytica* and *K. lactis* displayed the strongest extracellular proteolytic activity on skim milk agar, and none of the *D. hansenii* isolates showed any activity on this medium. *K. lactis* mainly produced acetaldehyde, ethanol, branched chain aldehydes and alcohols, and acetic acid esters, which were responsible for alcoholic, fruity and acetic notes. The volatile profiles of *D. hansenii* were rather limited and characterized by high levels of methyl ketones.

**Table 3.** Yeast species isolated from soft cheeses

Yeasts species	Cheese	Reference
*Candida inconspicua*	Bryndza	[72]
*Candida intermedia*	Manouri (whey cheese)	[73]
*Candida mogii*	Manouri (whey cheese)	[73]
*Candida xylopsoci*	Bryndza	[72]
*Debaryomyces hansenii*	Bryndza, Manouri (whey cheese)	[72],[73]
*Galactomyces candidus*	Bryndza	[72]
*Geotricum spp*.	Robiola di Roccaverano (acid coagulated)	[71]
*Kluyveromyces lactis*	Robiola di Roccaverano (acid coagulated)	[71]
*Kluyveromyces marxianus*	Bryndza, Water Buffalo Mozzarella	[72],[74],[75]
*Pichia farinose*	Manouri (whey cheese)	[73]
*Pichia kudriavzevii*	Bryndza	[72]
*Pichia membranefasciens*	Manouri (whey cheese)	[73]
*Saccharomyces cerevisiae*	Manouri (whey cheese), Water Buffalo Mozzarella, Cacio Cavallo Podolico	[73]–[75]
*Torulaspora delbrueckii*	Manouri (whey cheese)	[73]
*Trichosporon lactis*	Bryndza	[72]
*Yarrowia lipolytica*	Bryndza	[72]
*ZygosSaccharomyces rouxii*	Manouri (whey cheese)	[73]

Dolci et al. [67] studied the evolution of rind microflora in Fontina Protected Denomination of Origin semi-hard cheese. Yeasts were found to increase from 10^3^ to 10^6^ cfu/g in 28 days; a consequent rise of pH in the surface of cheese was observed (see bacteria surface ripened cheeses), together with an increase in the number of salt-tolerant bacteria, mainly coryneforms which reached 10^9^ cfu/g. *D. hansenii* and *Candida sake* were the species more constantly present throughout the whole maturing process.

Formagio di Fossa is a semi-hard cheese, with a special feature; the process of ripening occurs in special underground pits placed in a delimited area in the center of Italy [68]. Eight different yeast species were identified from pit environment: *C. zeylanoides*, *Candida norvegica*, *Pichia occidentalis*, *Pichia guilliermondii*, *Pichia jadinii*, *Cryptococcus albidus*, *Cryptococcus skinneri*, and *Sporobolomyces roseus*. Only *C. zeylanoides* was also found at the end of the maturation, together with the new isolated species *Wickerhamomyces anomalus*, *S. cerevisiae, D. hansenii*, and *Candida homilentoma* [68].

Dutch-type cheeses are semi-hard cheese varieties in which the few small eye-holes are formed by the CO_2_ produced from the fermentation of citrate by starter culture. Thus, citrate-positive bacteria are used as mesophilic starter culture. A special step in the cheesemaking is that the curd is washed with warm water, and cheeses are cooked at 37–45 °C, pressed, salted in brines and matured for 3–4 weeks to 1 year. Yeast counts in Gouda cheese showed an increase from 10^2^ cfu/g to 10^5^ cfu/g, but much slower than the lactic acid bacteria. The depletion of lactose is characteristic for Dutch-type cheeses, and the increase of yeasts after the depletion of lactose was occurred, possibly yeasts utilize the organic acids produced by the lactic acid bacteria, which consequently lead to an increase in pH. *Candida catenulata*, *C*. *laurentii*, *C*. *zeylanoides*, *C. albidus*, *D*. *hansenii*, *K*. *marxianus*, *Rhodotorula glutinis*, *R*. *minuta*, *S*. *cerevisiae*, *S. roseus*, *T*. *delbrueckii*, *T. beigellii* and *Y*. *lipolytica* were identified [70].

### 3.3 Soft cheeses

Soft cheeses are cheeses with soft texture, while some of them are spreadable; they are either coagulated with rennet or with acid, as the milk is coagulated from the acid at a pH of 4.6. Soft cheeses have a moisture content higher than 55%. The acidification is either caused by the adventitious lactic acid bacteria, or by the addition of an acid. Some soft cheeses are consumed fresh; some are ripened for 5–60 days [50]. The most important yeasts isolated from soft cheeses are shown in Table 3.

Bonetta et al. [71] studied the microbiological characterization of a typical Italian cheese Robiola di Roccaverano. Cheese samples were collected from four artisanal and one industrial producer. Artisanal producers used raw caprine milk and natural fermentation, whilst the industrial producer used mixed bovine-caprine milk and selected starters. The identification methods showed that microbial species such as *Geotricum* spp. and *K. lactis* that are related to the production of this typical cheese.

Bryndza cheese is a soft spreadable cheese, made from unpasteurized ovine milk. It is a traditional food product produced in mountain regions of Slovakia. May Bryndza cheese is a highly valued variant of Bryndza, which is produced in the beginning of summer season, in May. The diversity of yeasts encompassed *Candida xylopsoci*, *C. inconspicua*, *D. hansenii*, *Galactomyces candidus*, *K. marxianus*, *Pichia kudriavzevii*, *Trichosporon lactis* and *Y. lipolytica* [72].

A distinct category of soft cheeses are the whey cheeses. Whey cheeses are characterized by the fact that the coagulation of the milk (or whey) in caused by heating at 85–90 °C. Whey from the manufacture of hard or other cheeses is mixed with milk and/or cream and heated at 88–90 °C for 40–45 min; heating causes aggregation of the whey proteins and formation of the curd. Salt is added and the curd is moulded, drained and cooled [50]. The heating process causes killing of the yeast present in the curd and yeasts appear after post-heating contamination. There was a great diversity of yeast species, but *D. hansenii* and *Pichia membranefasciens* predominated. Other yeast strains found were *T. delbrueckii*, *Pichia farinosa*, *Candida mogii*, *Candida intermedia*, *Zygosaccharomyces rouxii*, *S. cerevisiae* [73].

Mozzarella is a soft pasta-filata cheese. Pasta-filata cheeses are manufactured from curd that are cooked at 60–70 °C, kneaded and stretched to form a smooth, plasticised, fibrous texture [50]. Some past-filata cheeses, such as Mozzarella di Bufala, are consumed fresh, and most of them are matured for 2–4 months, however, the maturation can be as long as up to 2 years for some cheese types (e.g., Caciocavallo Podolico). According to Romano et al. [74], the numbers and species of yeasts in the different cheeses vary, and there are certain yeast species that are more frequently detected than others. For instance, the galactose fermenting *S. cerevisiae* is often detected in pasta filata cheeses. Recently, a study has focused on lactose and/or galactose fermenting species *Kluyveromyces* and *Saccharomyces*, in order to evaluate their role on the functional and sensory properties of Mozzarella. The dominance of fermenting yeasts such as *K. marxianus* and *S. cerevisiae* suggests that these yeasts contribute to the development of the sensory characteristics of Mozzarella cheese [75].

### 3.4 White brined cheeses

White-brined cheeses constitute a separate family of cheeses, the characteristics of which is that they are ripened and preserved in brine until delivered to the customer. They are soft, semi-soft to semi-hard cheeses, traditionally produced under various names in the Balkans and Middle East and neighbouring countries. Traditionally, white brined cheeses are made mainly from ovine, caprine and buffalo milks, therefore, they retain the white colour of these milks. Mesophilic or thermophilic starter cultures are used and some varieties are cooked (e.g., the Greek Sfela and the Cypriot Halloumi), while most are not. The characteristic step in the cheesemaking process of these cheeses is that the maturation takes place with the cheese submerged in brine [50].

**Table 4.** Yeast species isolated from white brined cheeses

Yeasts species	Cheese	Reference
*Candida albicans*	Domiati	[78]
*Candida boidinii*	Halloumi	[76]
*Candida butyric*	Danish Feta-type	[76]
*Candida famata*	Feta, Brine	[76],[77]
*Candida krisii/zeylanoides*	Feta	[77]
*Candida parapsilosis*	Halloumi	[76]
*Candida sake*	Feta, Danish Feta-type	[76],[77]
*Candida sphaerica*	French Feta-type	[76]
*Candida versatilis*	Halloumi, Brine	[76]
*Clavispora lusitaniae*	Domiati	[76]
*Cryptococcus albidus*	Halloumi (bovine milk)	[78]
*Debaryomyces hansenii*	Feta, Danish Feta-type, Halloumi, Brine	[76],[77]
*Issatchenkia orientalis*	Domiati	[78]
*Kluyveromyces blattae*	French Feta-type	[76]
*Kluyveromyces lactis*	Feta	[77]
*Kluyveromyces marxianus*	Feta, Danish Feta-type, Brine, Domiati	[76]–[78]
*Kluyveromyces thermotolerance*	French Feta-type	[76]
*Kodamaea ohmeri (Pichia ohmeri)*	Domiati	[78]
*Pichia farinose*	Feta	[76]
*Pichia membranaefaciens*	Feta, Halloumi (ovine and bovine milk), Brine	[76],[77]
*Saccharomyces cerevisiae*	Feta, Brine	[76],[77]
*Torulaspora delbrueckii*	Feta, Danish Feta-type, Brine	[76],[77]
*Yarrowia lipolytica*	Danish Feta-type, Brine	[76]

The microbiology of white brined cheese has been reviewed [76]. More recently, Rantsiouet al. [77] examined the components of the microflora of four Feta cheeses, produced by different Greek manufacturers, using culture dependent and independent techniques. The main yeast species found were *S. cerevisiae*, *D. hansenii*, *C. famata*, *P. membranifaciens*, *T. delbrueckii*, *K. marxianus*, *Candida sake* and *K. lactis* [77].

Domiati is a well-known white-brined cheese; while fresh Domiati cheese is less salty and stored for a few weeks under refrigeration, Domiati cheese is highly salted and stored for a few months in brine solution or salted whey [78]. Identification results showed that the isolated yeasts belonged to the species *I. orientalis*, *Candida* albicans, *Clavispora lusitaniae* (*Candida lusitaniae*), *Kodamaea ohmeri* (*Pichia ohmeri*), *K. marxianus*, and *Candida catenulata*. These identified yeasts were recovered from samples of all examined products, however, different degrees of yeast diversity were observed in these products. While fresh Domiati cheese contained a single but different yeast species, matured Domiati contained a diverse range of yeast species. Total yeast counts in Domiati cheese were generally higher than 10^3^ cfu/g, but lower than 10^5^ cfu/g [76]. It is worth noting that *C. arbicans* was isolated from fresh Domiati cheese. *C. arbicans*, together with *Candida parapsilosis*, *Candida tropicalis* and *Candida guillermondii*, also known as the most common yeast pathogens, are opportunistic commensals responsible for various mycoses [79],[80]. However, none of these species are found in mature cheese, and only found in fresh cheese and brines [81] and are probably unable to survive the maturation process [79].

### 3.5 Mould surface ripened

Mould surface ripened cheese varieties have a special characteristic, that is the growth of *Penicillium camemberti* on the surface of the cheese, causing the characteristic softening of the cheese. During the first phase, after the by a mesophilic starter, the pH is below 5.8, only an acidophilic flora, that is yeasts, such as *D. hansenii*, *K. lactis*, *G. candidum*, which, together with the mould *P. camemberti* raise the pH by consuming the lactate for their growth [82]. At the same time, lactate migrates from the core, and calcium phosphate precipitates and soluble calcium phosphate migrates to the surface. During the second phase, bacteria adapted to the high salt content of the cheese such as staphylococci or coryneforms will start to grow and contribute to the maturation process [50].

The main yeasts found in mould surface ripened cheeses are *D. hansenii*, *K. lactis*, *K. marxianus* and *G. candidum*. Other species such as *S. cerevisiae*, *Y. lipolytica* and *Candida* spp. are occasionally present. *K. lactis* and *K. marxianus* metabolise residual lactose first; when lactose has exhausted, lactate will be metabolised by *D. hansenii* and other yeasts. The pH of mould surface ripened cheeses increase slowly during the first 5 days, but the growth of *P. camemberti* cause a very fast increase in pH at the surface. The pH increases from less than 5 to 7.5 in less than 2 days. Yeast population numbers were found to be greater than 10^6^ cfu/g in the surface of Camembert cheese [83]. The most predominant species isolated were *D. hansenii*, *Candida catenulata*, *C. lipolytica*, *C. kefyr*, *C. intermedia*, *S. cerevisiae*, *Cryptococcus albidus* and *K. marxianus*. In another study, Camembert and Brie cheeses were monitored for their yeast populations in order to determine the seasonal diversity of yeasts [84]. It was found that yeasts play a significant role during maturation. *D. hansenii* and *Y. lipolytica* were the most abundant yeast species isolated from Camembert and Brie cheeses. In addition, *T. delbrueckii*, *Rhodotorula mucilaginosa*, *Rhodotorula minuta* and various species of *Candida* were also isolated.

Addis et al. monitored the growth of yeasts and bacteria during the maturation of Australian Camembert cheese [85]. Yeasts reached 10^5^–10^9^ cfu/g, throughout the maturation, depending on the manufacturer. *D. hansenii* predominated, and *Y. lipolytica* were present. Interactions between the various yeasts and bacterial isolates were examined [85].

**Table 5.** Yeast species isolated from mould surface ripened cheeses

Yeasts species	Cheese	Reference
*Candida*.spp.	Camembert and Brie	[83]
*Debaryomyces hansenii*	Camembert and Brie	[83],[84]
*Rhodotorula minuta*	Camembert and Brie	[83]
*Rhodotorula mucilaginosa*	Camembert and Brie	[83]
*Torulaspora delbrueckii*	Camembert and Brie	[83]
*Yarrowia lipolytica*	Camembert and Brie	[83],[84]

Chen et al. [86] examined the influence of selected yeast strains on Camembert-type cheeses. Yeast population grew exponentially and then slowed to a moderate growth rate throughout maturation. The reported results indicated that the selected strains had a significant effect on the content and ratio of individual free amino acids, and thus on the development of flavor, whereas the addition of adjunct culture had no effect on the lipolysis. In the cheese with added *I. orientalis*, a greater amount of small peptides and a higher concentration of non-protein nitrogen and NH_3_ were found.

### 3.6 Bacterial surface ripened

Bacterial surface ripened, also called smear cheeses, are characterized by the development of special bacterial microflora (smear) on the surface of the cheese. Some varieties are curd washed and cooked, depending on the fat content, and moulded. Some are pressed and salted in brine. Throughout the maturation, the surface of the cheese is washed with a brine solution containing special bacterial microflora. The milk is coagulated with rennet and then a diverse group of yeasts (see Table 6) begin to grow on the cheese surface. The yeasts are metabolizing the residual lactate, producing CO_2_ and H_2_O and causing thus, an increase in the pH. In addition, deamination of amino acids and production of NH_3_ may occur. This deacidification favours the growth of a complex bacterial microflora, and yeasts produce compounds that stimulate the bacterial growth. This microflora includes various coryneforms (e.g., *Corynebacterium* spp., *Arthrobacter* spp. and *Brevibacterium* spp.), micrococci and staphylococci. Some of these organisms are pigmented, which leads to the characteristic red-orange colour of smear cheeses [50],[87]. Although considerable variation occurs, the most prevalent yeasts reported in bacterial surface ripened cheeses include *D. hansenii*, *Candida* spp., *Trichosporon* spp., *Y. lipolytica*, *Kluyveromyces* spp., *Rhodotorula* spp. and *Torulaspora* spp., together with *G. candidum* [88].

Dugat-Bony et al. [89] investigated the specificity and diversity of cheese microbiota associated with 60 cheeses belonging to 12 traditional French cheese varieties, that is Epoisses, Maroilles, Soumaintrain, Saint-Marcellin, Langres, Munster, Livarot, Pont l' Eveque, Mont d' or, Abbaye de Giteaux, Reblochon and Saint-Nectaire, manufactured from bovine milk. Bacterial surface ripened cheeses host complex microbial communities responsible for the transformation of milk into cheese as well as the development of important properties in terms of texture, color and sensory perception, and, little variation was observed regarding the fungal community composition over cheese varieties and sample types (rind or core) [89]. Indeed, most samples were dominated by *G. candidum*, *D. hansenii* and *C. sake*, with minor detection of other fungal species.

The composition of microbial communities varies according to the cheese variety as seems from the results obtained by Quigley et al. [49] for a variety of Irish cheeses. Wolfe et al. [48] stated that the rind type and parameters such as moisture together with certain cheesemaking manufacturing steps, such as the coagulation type (lactic vs rennet) or the draining method, have a great impact on the cheese microbial communities. Although a positive correlation was observed between bacterial diversity and pH, this was not observed for fungi, that are generally known to be tolerant to the acidification [90].

**Table 6.** Yeast species isolated from bacteria surface ripened cheeses

Yeasts species	Cheese	Reference
*Candida sake*	Smear cheese, Taleggio, Danish surface-ripened cheese	[89],[92],[93]
*Debaryomyces hansenii*	Danish surface-ripened cheese	[92]
*Geotrichum candidum*	Smear cheese	[89]
*Geotrichum* spp.	Danish surface-ripened cheese	[92]
*Kluyveromyces lactis*	Taleggio	[92]
*Kluyveromyces marxianus*	Taleggio	[92]
*Pichia guilliermondii*	Taleggio	[92]
*Torulaspora delbruecki*	Taleggio	[92]
*Yarrowia lipolytica*	Taleggio, Danish surface-ripened cheese	[92],[93]

Production of bacteria surface ripened cheeses depends on the surface growth of a diverse group of bacteria and yeasts. These microorganisms often originate from the cheesemaking facility forming interesting and complex associations [91]. While commercial adjunct cultures are frequently used, it is not clear enough whether these strains are able to establish successfully within the resisting microflora. Goerges et al. studied the fate of adjunct cultures in Limburger cheese; the cheese was supplemented with a commercial adjunct culture containing *D. hansenii*, G. *Geotrichum*, *Arthrobacter arilaitensis*, and *Brevibacterium aurantiacum* [91]. While certain yeast present in the culture (i.e. *D. hansenii*), the bacterial smear cultures could not be reisolated from the cheese surface at all. Goerges et al. concluded that none of the adjunct bacterial strains were able to compete significantly against the resident microbial consortia [91].

The yeast microflora of four Danish bacterial surface ripened cheeses produced at three farmhouses and one industrial dairy was investigated [92]. The surface yeast microbiota consisted primarily of one dominating species for each cheese. *Y. lipolytica*, *Geotrichum* spp. and *D. hansenii* were the dominant species for the farmhouse cheeses, while in the industrially manufactured cheese, only *D. hansenii* was isolated.

Taleggio is a soft bacterial surface ripened cheese, produced in Northern Italy by pasteurized milk. The maturation of Taleggio is a complex and dynamic process, influenced by natural microflora and the addition of selected lactic acid bacteria starters; it is not inoculated with commercial yeast starter cultures. The microbial groups involved in the maturation are mesophilic bacteria, micrococci, staphylococci, coliforms, thermophilic streptococci, lactobacilli, yeasts, and moulds [93]–[95]. *G. candidum* and *D. hansenii* were the dominant yeasts, and these were implicated in various stages of the maturation process; *K. marxianus* and *K. lactis* were isolated in small frequencies [95]. Sequence analysis of isolates brought to the identification of six frequent species: *D. hansenii*, *K. lactis*, *K. marxianus*, *Y. lipolytica*, *Pichia guilliermondii*, and *T. delbrueckii*, and two additional species *C. sake* and *Candida etchellsii* [96].

### 3.7 Blue cheeses

Blue cheeses are characterized by blue veins caused by the growth of *Penicillium roqueforti* in the interior of the cheese. The acidification is carried out by mesophilic lactic starter cultures and the milk is coagulated by rennet extracts. The curd is cut at the size of hazelnut to walnut and, for some varieties, are washed with warm water before being transferred in the moulds and after that they are dry-salted. The curd is not pressed, since the growth of the mould needs oxygen, and thus an open texture. The curds are pierced with needles containing mould spores. Blue cheeses have a strong flavour, caused by the extensive lipolysis, and the presence of n-methyl-ketones which are produced from fatty acids [50].

Yeast microflora on the surface and interior of Rokpol, a Polish blue cheese, was investigated; yeast populations on the surface of the cheeses ranged from 10^5^–10^9^ cfu/g, but were 10–100 times lower for interior samples, showing a great variability. The most frequently isolated species were *C. famata* and *C. spherica*, followed by *C. intermedia* and *Geotrichum* ssp. Other species such as *Saccharomyces kluyveri*, *C. kefyr* and *C. lipolytica* were found occasionally [97].

Significant qualitative and quantitative differences were observed in the yeast communities between the cheese sections of Stilton cheese. *Y. lipolytica* presented strong synergistic activity with *P. roqueforti* enhancing the production of ketone aroma compounds, characteristic of blue cheeses. *Y. lipolytica* was present in the white parts, where the *P. roqueforti* was also present but existed in the mycelial form. *K. lactis* dominated in the blue veins, was less present in the white core, and had limited presence in the outer crust and thus followed a similar distribution pattern with the *P. roqueforti*. It is interesting to note that *Y. lipolytica* and *P. roqueforti* isolates when grown together delivered enhanced ketone production typical of Stilton aroma. *Y. lipolytica* strains synergistic with the starter *Penicillium* which do not inhibit its sporulation would have excellent potential as starter cultures [97].

The most frequently occurring yeasts on the exterior of Gorgonzola-type blue cheese were species of *D. hansenii*, *C. versatilis*, *T. beigelii* and *T. delbrueckii* [98]. In the interior, an enhanced diversity in the yeast population was obtained. *D. hansenii* clearly predominated on the exterior and in the interior of the cheese represented by more than 30% of the population. *C. versatilis* was the second most abundant yeast species on the exterior and in the interior of the cheese, whereas, *T. delbrueckii* strains were also frequently encountered. *S. cerevisiae*, *Rhodotorula glutinis*, *C. zeylanoides* and *Cryptococcus albidus* were only found in the interior of the Gorgonzola-style blue cheese. Species recovered from Gorgonzola- and not from Danish-style, were *T. beigelii*, *R. glutinis* and *C. zeylanoides* [98]. In Kopanisti cheese, where *P. roqueforti* is a main part of the microflora, *T. ovoides* was found to be the dominant yeast species [99].

*D. hansenii* is one of the most frequently reported yeasts in blue cheeses [85],[95],[100]. For this reason, and because of its strong proteolytic activity it has been suggested that it should be used as part of commercial adjunct cultures. The species was previously reported to stimulate the growth and sporulation of *P. roqueforti* isolates from blue cheese [101]. However, the phenomenon was strain specific and *D. hansenii* was found to be the dominant yeast species of both good and poor blue veined cheeses [101].

**Table 7.** Yeast species isolated from blue-veined cheeses

Yeasts species	Cheese	Reference
*Candida catenulate*	Stilton	[97]
*Candida famata*	Rokpol	[96]
*Candida intermedia*	Rokpol	[96]
*Candida kefyr*	Rokpol	[96]
*Candida lipolytica*	Rokpol	[96]
*Candida spherica*	Rokpol	[96]
*Candida versatilis*	Gorgonzola-type	[98]
*Candida zeylanoides*	Gorgonzola-type	[98]
*Cryptococcus albidus*	Gorgonzola-type	[98]
*Debaryomyces hansenii*	Stilton, Roquefort, Mycella, Gorgonzola-type	[97],[98],[100],[102]
*Geotrichum* ssp.	Rokpol	[96]
*Rhodotorula glutinis*	Gorgonzola-type	[98]
*Saccharomyces cerevisiae*	Mycella	[102]
*Saccharomyces kluyveri*	Rokpol	[96]
*Torulaspora delbrueckii*	Gorgonzola-type	[98]
*Trichosporon beigelii*	Gorgonzola-type	[98]
*Trichosporon ovoides*	Stilton, Kopanisti	[97],[99]
*Yarrowia lipolytica*	Stilton	[97]

The potential use of *S. cerevisiae* FB7 as an additional starter culture for the production of Mycella, a Danish Gorgonzola-type cheese, was investigated [102]. Two dairy productions of Mycella, each containing batches of experimental cheeses with *S. cerevisiae* added and reference cheeses without yeast added were carried out. While *D. hansenii* dominated in the reference cheese and on the surface of the experimental cheeses. In the cheeses with *S. cerevisiae* FB7, an earlier sporulation and an improved growth of *P. roqueforti* was observed compared to the reference cheeses. Furthermore, in the experimental cheese, synergistic interactions were also found in the aroma analysis, the degradation of casein and by the sensory analysis. The observed differences indicate a positive contribution to the overall quality of Mycella by *S. cerevisiae* FB7 [102].

### 3.8 Brines

Brines used for salting the cheese have been reported to be an important source of yeasts. Together with the chemical composition, mainly salt content, the brine microflora has a great impact on the microbiology of the cheese used for and, consequently, on the quality characteristics of the cheese employed [103]. However, limited knowledge is available on the occurrence of microorganisms in cheese brine.

*C. intermedia*, *D. hansenii*, *K. lactis*, *Papiliotrema flavescens*, which have not been isolated and identified from dairy industry-related products, *R. glutinis*, *Sterigmatomyces halophilus* and *Yamadazyma triangularis*, formerly named *Pichia triangularis*, were isolated and identified from Danish brines used for surface-ripened semi-hard Danbo cheeses production [30]. In addition, Zhang et al. studied the effects of salt and temperature on growth and survival of yeasts from brines and concluded that the yeast strains tested could grow in conditions similar to the cheese surface [104]. The cheese brine mimicking condition, at low temperature was found to have a more beneficial for growth of the yeast strains at high NaCl concentrations, whereas a higher temperature is more favorable at low NaCl concentrations [104].

Salting by brining has an important effect on the structure and flavour of the cheeses and is significant in regulation of the microbiota on the cheese for a number of cheese varieties, such as surface ripened cheeses [105]–[108] and white-brined cheeses [109],[110].

However, despite the fact that brining is an important step in the production of a huge variety of cheeses, few detailed studies are available and limited focus has been placed on identification of eukaryotes in cheese brines. Knowledge on the brine microbiota could lead to a more comprehensive understanding of the establishment of the microbiota at the cheese surfaces. New procedures should be developed for brine handling including use of specifically adapted microbial cultures to control the brine microbiota and thereby the microbiota on the cheese surfaces in the early ripening period. Brine purification methods, such as chemical treatments with antimicrobials and preservatives, heat treatment, treatment with UVA and UVC light, filtration and microfiltration methods that are used in the dairy industry have been reviewed [103].

## 4. Defects

Yeasts, when present in high counts on the surface of certain cheese can cause early gas formation, that is gas creating numerous small holes and produced in cheeses shortly after manufacture [111]. Gas caused by yeasts is CO_2_ produced from metabolism of lactate or lactose. In white brined cheese, early gas may cause blowing of the cheese block or swelling of the cheese containers, and yeasts that may be involved in early gas blowing include *K. lactis*, *Dekkera anomala* and *T. delbrueckii*, depending on the local factory; microflora and species vary from country to country [112]–[114]. Excessive yeast growth will cause softening of cheese, a condition that is usually associated with an unpleasant yeasty or ester-like odour [81]. Other defects are pink discolouration [115] and brown spots [116]. The formation of brown pigments, shown on the cheese surface, is caused by excessive growth of *Y. lipolytica* [24]. Discoloration of the surface of a Portuguese ovine cheese has been attributed to pigment producing *Y. lipolytica*, which enables brown pigments to be produced from tyrosine [117]. It is interesting to note that these brown pigments, which are formed from the catabolism of tyrosine have been reported to exhibit antimicrobial activity [118].

In addition, yeasts can cause deacidification at the surface of the cheese when they catabolize amino acids to produce NH_3_ [119]. The increase in the pH of the cheese, may be either from the utilization of lactates or from the production of NH_3_. In any case, the pH increase can spur the growth of *Staphylococcus aureus* [120] and possibly other pathogenic and/or spoilage bacteria with low acidic tolerance [48].

The production of biogenic amines, that is, toxic metabolites that produced from the decarboxylation of free amino acids. This reaction is caused by microbial enzymic activity and the most frequently produced biogenic amines are: histamine, tyramine, cadaverine, putrescine, tryptamine and phenylethylamine [24]. Although biogenic amines are produced from the metabolic action of certain lactic acid bacteria, yeast species such as *Y. lipolytica* and *D. hansenii* may have the potential to produce biogenic amines, but this trait is strain specific [21].

## 5. Concluding remarks

Yeasts constitute an important part of cheese microflora for many types of cheese. Over the past 15 years, knowledge of yeast diversity in cheeses has increased considerably, due to the use of molecular methods for identification and strain typing. Selected yeast species have been used as adjunct cultures for certain cheese types as have shown to contribute to the development of the special sensory characteristics during the manufacture and maturation [119].

The yeasts, usually coming as contaminants, are easily developed on the surface of the cheese and then, depending on the cheese type and their characteristics, contribute to the maturation process with their proteolytic and lipolytic enzymes. This contribution, which has not fully studied for each cheese type, depends on their population numbers and the specific yeast species and strains present. It appears that the prevailing yeast species in the most of cheese types is *D. hansenii* (Tables 1–7). *D. hansenii* has been identified in all seven categories; this prevalence may be due to its high halotolerance, since it can survive up to 20–24% (w/w) NaCl, together with their ability to grow at low pH and utilize lactate as the main carbon source [21]. This species can be found on the surface of a great number of cheeses and in the brines used for their salting, and brining step is the most probable contamination point. In addition to the hard cheeses shown in Table 1, where yeasts were isolated at some point during the manufacture, Banjara et al. isolated *D. hansenii* species from retail samples of Asiago, Gruyere, Parmesan-type, Romano and other hard cheeses [121].

*K. marxianus* and *K. lactis* were also abundant in most of the cheese types (Tables 1–4 and 6); this is due to their ability to utilize lactose as a source of carbon. These species are able to ferment lactose and produce ethanol and CO_2_, whereas, they can metabolize lactate, but after lactose is depleted [21]. *K. marxianus* and *K. lactis* have shown exceptional biochemical activities with proteolytic and lipolytic action, and also esterase activity producing esters and acetaldehyde, contributing thus, to the maturation and the development of flavour compounds. *G. candidum* is usually present in mould and bacteria surface ripened cheeses, but it has been isolated from retail samples of many hard cheeses [121]. *Y. lipolytica* is also a popular yeast in many cheese types (Tables 1, 2 and 4–7), and is characterized by its very strong proteolytic and lipolytic activities, producing great amounts of volatile compounds, contributing, thus, to the development of aroma and flavour of cheese [108]. However, the strong enzymic activity of *Y. lipolytica* may lead to off-flavours.

Besides these species, a great diversity of yeast species has been observed. For some cheese types yeasts are the main microbial group, at least for some part of their maturation process, while for some other types yeasts are absent. Differences between industrially manufactures cheeses and artisanal cheeses have specified. Artisanal cheeses possess a diverse assortment of yeast species, mainly belonging to the genera *Candida*, *Clavisporalus*, *Cryptococcus*, *Debaryomyces*, *Geotrichum* (=*Galactomyces*), *Issatchenkia*, *Kazachstania*, *Kluyveromyces*, *Kodemaea*, *Pichia*, *Rhodotorula*, *Saccharomyces*, *Saturnispora*, *Torulaspora*, *Trichosporon*, *Yarrowia* and *Zygosaccharomyces*. However, the prevalence of the yeast species are dependent on the specific characteristics of each cheese variety and the manufacturing steps followed during the manufacture. In addition, yeast species composition changes greatly along the cheese maturation process. The microflora in certain cheese types (e.g., white brined cheeses, surface-ripened and blue-veined cheeses) is complex and contains a broad, diverse range of yeasts with a special role in the maturation process. The great variability in cheesemaking process together with the variable maturation conditions makes the cheese ecology a diverse environment for the development of a great number of different microbial.

The knowledge of the whole cheese microbiome is a requirement in order to have a picture of the way the flavour and other sensory characteristic of each cheese variety is developed and to have a control of the overall quality of cheese. However, this knowledge needs to be integrated with the biochemical activities of the yeast strains and the numbers of the population present in the cheese throughout the maturation. In addition, the source of contamination, that is the source of these yeasts needs to be determined, in order to understand the impact of yeasts in the maturation process of each cheese type. Monitoring and controlling the level of contamination and identifying the yeast strains involved, as part of the whole cheese microbiota (i.e., together with the starter and non-starter lactic acid bacteria and other moulds) will give a picture of how the specific cheese sensory characteristics are developed for each type of cheese.

Species identification and characterization, together with the interactions with other microbial groups at specific stages of cheesemaking, are therefore essential to understand the role of yeasts in cheese.
